# Revolutionizing Healthcare: Qure.AI's Innovations in Medical Diagnosis and Treatment

**DOI:** 10.7759/cureus.61585

**Published:** 2024-06-03

**Authors:** Esteban Zavaleta-Monestel, Ricardo Quesada-Villaseñor, Sebastián Arguedas-Chacón, Jonathan García-Montero, Monserrat Barrantes-López, Juliana Salas-Segura, Adriana Anchía-Alfaro, Daniel Nieto-Bernal, Daniel E Diaz-Juan

**Affiliations:** 1 Pharmacy, Hospital Clínica Bíblica, San Jose, CRI; 2 Research, Hospital Clínica Bíblica, San Jose, CRI; 3 Pharmacy, Universidad de Iberoamerica, San Jose, CRI; 4 Cardiology, Hospital Clínica Bíblica, San Jose, CRI; 5 General Practice, AstraZeneca, San Jose, CRI; 6 Radiology, Hospital Clínica Bíblica, San Jose, CRI

**Keywords:** x-rays, ai, qureai, qxr, diagnosis, pulmonary nodules, heart failure, deep learning, artificial intelligence

## Abstract

Qure.AI, a leading company in artificial intelligence (AI) applied to healthcare, has developed a suite of innovative solutions to revolutionize medical diagnosis and treatment. With a plethora of FDA-approved tools for clinical use, Qure.AI continually strives for innovation in integrating AI into healthcare systems. This article delves into the efficacy of Qure.AI’s chest X-ray interpretation tool, "qXR," in medicine, drawing from a comprehensive review of clinical trials conducted by various institutions.

Key applications of AI in healthcare include machine learning, deep learning, and natural language processing (NLP), all of which contribute to enhanced diagnostic accuracy, efficiency, and speed. Through the analysis of vast datasets, AI algorithms assist physicians in interpreting medical data and making informed decisions, thereby improving patient care outcomes.

Illustrative examples highlight AI's impact on medical imaging, particularly in the diagnosis of conditions such as breast cancer, heart failure, and pulmonary nodules. AI can significantly reduce diagnostic errors and expedite the interpretation of medical images, leading to more timely interventions and treatments. Furthermore, AI-powered predictive analytics enable early detection of diseases and facilitate personalized treatment plans, thereby reducing healthcare costs and improving patient outcomes.

The efficacy of AI in healthcare is underscored by its ability to complement traditional diagnostic methods, providing physicians with valuable insights and support in clinical decision-making. As AI continues to evolve, its role in patient care and medical research is poised to expand, promising further advancements in diagnostic accuracy and treatment efficacy.

## Introduction and background

Over the years, technology has advanced remarkably, hand in hand with science. One of the great milestones of this progress was the emergence of artificial intelligence (AI) in 1956. Since then, AI has evolved by leaps and bounds, with increasingly novel advances in recent decades [1]. AI is defined as a network of systems capable of performing tasks or processes that normally require human intelligence, facilitating and automating the execution of different processes. This tool is based on algorithms that allow machines to learn data to have better performance in the future [2].

Qure.AI is a healthtech startup employing AI to assist in medical imaging diagnostics. The company was founded in 2016 and is based in Mumbai, Maharashtra, India. They develop deep-learning solutions designed to support physicians in routine diagnosis and treatment, enabling them to dedicate more time to patient care. Qure.AI’s qXR is a software application capable of using deep learning algorithms to detect nodules on chest X-rays. Additionally, qXR helps in the early detection of heart failure on chest X-rays by analyzing and interpreting abnormalities in medical imaging outputs [3]. With a robust emphasis on improving accessibility, affordability, and timeliness of care, Qure.AI has made considerable progress in reshaping the healthcare environment across various global contexts [4].

In Africa, Qure.AI has been instrumental in tuberculosis (TB) screening, utilizing its AI algorithms to accurately and efficiently identify TB cases. By facilitating early detection and timely initiation of treatment, Qure.AI has helped alleviate the burden of TB in the region, leading to saved lives and enhanced public health outcomes [5].

In Vietnam, Qure.AI's AI-driven system for detecting lung cancer has shown impressive efficacy. Through the analysis of medical images and the identification of potential lung cancer cases, the company has enabled early intervention, resulting in enhanced survival rates. This example highlights the significant potential of AI in addressing pressing health issues and advancing cancer treatment, particularly in regions with limited resources [6].

Qure.AI's impact extends to developed nations, particularly the United States, where its AI-driven stroke management solutions have demonstrated promising outcomes. These innovations have enhanced treatment strategies and patient outcomes by empowering healthcare professionals to rapidly and precisely assess stroke incidents. This capability facilitates timely decision-making and targeted interventions, ultimately reducing the negative impact of strokes [4].

The Qure.AI Impact Report highlights the company's strategic partnerships with healthcare institutions, government bodies, and industry stakeholders, which have greatly expanded the reach and impact of its AI solutions while fostering innovation. Additionally, the report showcases Qure.AI's global growth in AI implementation, reflecting the increasing recognition of AI's value in healthcare. It emphasizes the company's commitment to regulatory compliance and quality assurance, ensuring that its AI solutions meet the highest standards of safety and efficacy and underscoring Qure.AI's dedication to responsible and trustworthy technology [4].

In addition, a positive impact on expenses has also been reflected with the use of AI technology, which shows a reduction in costs in medical processes, effective times, and early therapeutic approaches, among others. This advantage is complemented by a decrease in hospital readmissions, which is achieved through predictive models enabled by this technology, which enhance the accuracy of initial diagnoses and treatments [7].

AI has had a great impact on the clinical field, providing benefits that significantly improve the results of procedures previously performed exclusively by humans. Greater assertiveness and effectiveness in the results have been observed, reducing human errors when doctors work jointly with this technology. Likewise, it has been proven that by using resources like these, the generation of results is accelerated, such as diagnosis and timely treatment, among others. The purpose is for AI to become a second opinion for health professionals when making decisions or reading diagnostic results [8].

## Review

Methodology

To demonstrate the effectiveness and contributions of using Qure.AI’s chest X-ray interpretation tool, "qXR," in the healthcare field, a search was conducted through various sources. Literature from databases such as PubMed, Google Scholar, ClinicalKey, and even various medical journals was reviewed. Articles considered to have a complete set of information were obtained from scientific sources. In the case of those dealing with clinical studies, publications between 2018 and 2024 were considered. In addition, the clinical studies analyzed have also shown the effectiveness of AI in the diagnosis and interpretation of X-rays, especially involving heart failure (HF) and lung condition cases, such as the appearance of nodules. All images used in this article are either created by the authors or obtained from licensed sources. The authors hold the right to use these images for publication in this specific article.

Results

Table 1 shows different clinical studies of the effectiveness of Qure.AI, considered as the main evidence base for this review, as these studies demonstrate the use of AI in the health field and its respective results [9-14].

**Table 1:** Comparison of clinical studies showing the effectiveness of Qure.AI’s chest X-ray interpretation tool (qXR) CTR: Cardiothoracic ratio.

Author	Year	Title	Type of the study	Number of patients	Results
Singh et al. [12]	2018	Deep learning in chest radiography: detection of findings and presence of change	Clinical trial	724 patients	Various abnormalities were detected in 58% of the X-rays.
Ahmad et al. [13]	2018	Machine learning methods improve prognostication, identify clinically distinct phenotypes, and detect heterogeneity in response to therapy in a large cohort of heart failure patients.	Clinical trial	44,886 patients	Cases of heart failure were identified with the help of four different algorithms that helped in the early diagnosis of the problem.
Celik et al. [14]	2022	The early diagnostic value of chest X-ray scanning by the help of artificial intelligence in heart failure (ART-IN-HF): the first outcomes	Clinical trial	5623 patients	Cardiomegaly and pleural effusions were diagnosed in X-rays.
Mahboub et al. [10]	2022	Identifying malignant nodules on chest X-rays: a validation study of radiologist versus artificial intelligence diagnostic accuracy	Clinical trial	213,459 patients	It provided 96%-99% specificity and 95%-100% sensitivity in diagnosis.
Govindarajan et al. [11]	2022	Role of an automated deep learning algorithm for reliable screening of abnormality in chest radiographs: A prospective multicenter quality improvement study.	Clinical trial	65,604 X-rays	It obtained an accurate prediction of 98.9% for abnormalities such as nodules, cardiomegaly, and fibrosis.
Celik et al. [9]	2023	The diagnostic value of chest X-ray scanning by the help of artificial intelligence in heart failure (ART-IN-HF)	Clinical trial	10,000 patients	Increased CTR and pleural effusions were detected.

As demonstrated in Table 1, numerous international studies highlight the effectiveness of using Qure.AI’s qXR in the healthcare field. These studies, which involve a large group of patients, demonstrate promising diagnostic results due to the supportive function provided by this technology to medical professionals and healthcare institutions. Additionally, the studies indicate improvements in the speed, specificity, and accuracy of diagnostic results. The positive outcomes from these studies enhance the credibility of Qure.AI systems, leading to their increased adoption in health centers worldwide.

Discussion

AI applies various techniques, and each one plays a distinctive and beneficial role in the healthcare field. Machine learning is one of the most common AI techniques. It allows models to "learn" from data. Its application is aimed at predicting which treatment protocols are most likely to be effective for a patient, taking into account various patient attributes and the treatment context [15].

As for deep learning, it can detect patterns; so, it is used to recognize possible lesions such as neoplasms in X-rays. It is progressively being used in radiomics, which involves identifying clinically significant features in imaging data that go beyond human visual perception. Both radiomics and deep learning are predominantly utilized in oncology-focused image analysis. The integration of these technologies seems to offer greater diagnostic accuracy compared to earlier automated image analysis tools [15].

**Figure 1:**
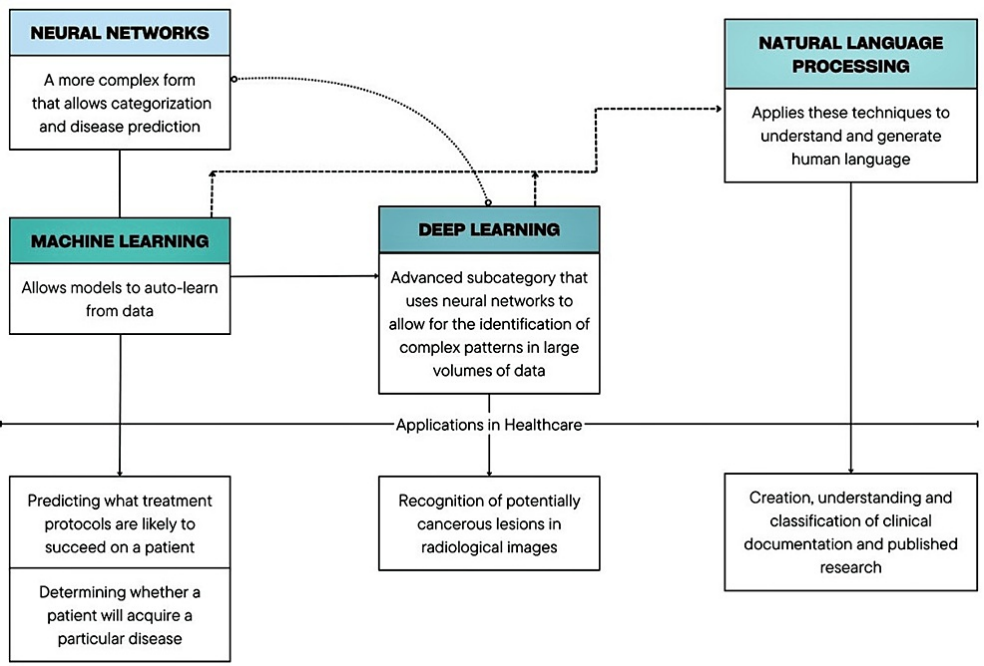
Relationship between AI techniques and their applications in healthcare Image credits: This image was created by the authors.

Furthermore, the field of natural language processing is a branch of AI that focuses on understanding and generating human language. It uses machine learning techniques, especially deep learning, for tasks such as voice recognition, text analysis, and translation. In healthcare, NLP is employed for analyzing unstructured clinical notes, producing reports, and transcribing patient interactions, thus enhancing the effectiveness and precision of clinical documentation and diagnosis. Because of NLP, it is possible to contribute to the statistical organization of patients’ data in hospitals and healthcare centers [15]. Figure 1 shows the relationship between the most used techniques in the healthcare setting [15].

AI has demonstrated that when used in the interpretation of X-rays, results can be obtained in approximately one minute, while computed tomography (CT) scans take around 10 minutes. This proves that AI is an efficient tool and can assist physicians in expediting the diagnostic process [16].

**Figure 2:**
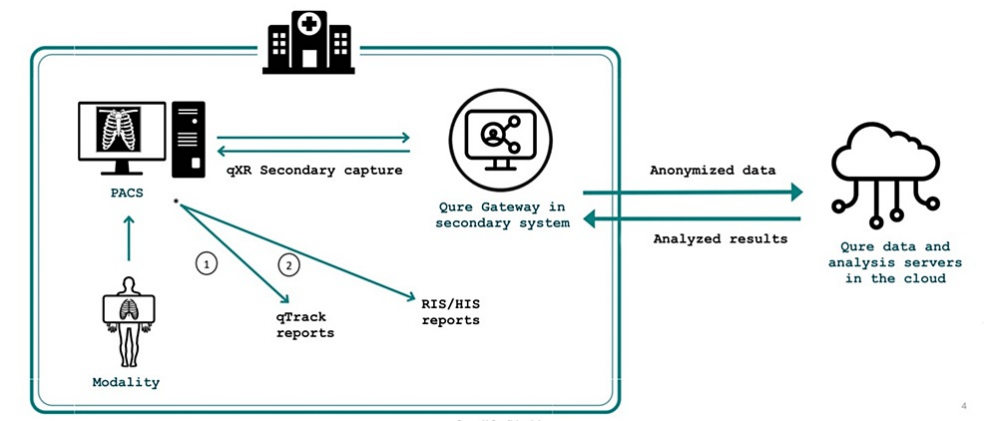
Patient data flow process and AI data installation in the cloud This graphic illustrates the flow of information in AI-assisted analysis of chest radiographs. Initially, the patient undergoes a chest X-ray, captured by radiological equipment. The radiograph data is then transferred to a computer for initial image processing. Subsequently, data (anonymized) is transmitted to the cloud, housing the AI system for detailed analysis. Using advanced algorithms, the AI examines the radiograph to detect potential lung pathologies, generating a comprehensive report. Finally, the AI-generated report is sent back to the radiologist's computer, aiding in clinical diagnosis. This integrated AI-driven radiological analysis enhances accuracy and efficiency in the early detection of lung pathologies. Image credits: Permission was obtained from the original publisher to adapt this image [16]. PACS: Picture Archiving and Communication System; RIS: Radiology Information System; HIS: Hospital Information System.

It is important to note that there exists a specific process enabling AI to operate on radiology equipment, facilitating rapid and accurate generation of results. This process entails information being processed, stored in the cloud, and subsequently utilized by the equipment to generate results (Figure 2) [16].

AI has made significant contributions to healthcare treatments, particularly in the areas of intervention selection, medication safety, and adverse event management. AI's ability to generate personalized treatment options based on a patient's genetics, biomarkers, and lifestyle has revolutionized healthcare. For pharmacists, AI can be a powerful tool to enhance treatment safety for patients and achieve better overall therapeutic outcomes [17,18].

AI also plays a crucial role in identifying and predicting data patterns to anticipate events that could potentially harm patients or healthcare institutions. This proactive approach can lead to reduced treatment costs, lower patient expenses associated with delayed diagnosis or treatment, and improved resource allocation within healthcare systems. Additionally, AI-powered predictive analytics can identify patients at the risk of developing chronic diseases, enabling early intervention and prevention strategies to avert future complications, readmissions, and associated costs [19].

The primary focus of this article is the use of AI, specifically Qure.AI’s chest X-ray, where accurate interpretation of X-rays can be complex and difficult to achieve. In the field of diagnosis, AI has garnered increasing interest due to its ability to improve the speed, accuracy, and efficiency of radiologists' interpretation of results. Qure.AI’s tools are intended to serve as a complementary resource for physicians in interpreting X-rays and generating diagnoses, offering valuable support in clinical decision-making [20].

As mentioned earlier, Qure.AI has been applied in multiple contexts within the clinical setting. However, when focusing on its diagnostic function, several examples of its usefulness stand out. One area where AI has proven to be of great assistance to medical professionals is in the early diagnosis of HF [9]. This condition arises because of an alteration, either structural or functional, in the heart, which causes an increase in intracardiac pressure and an inability to pump an adequate amount of blood to meet the body's demands, both at rest and during exercise [21].

HF produces a series of symptoms in the patient, which may lead a physician to suspect the condition, such as shortness of breath, fatigue, irregular heartbeat, and wheezing, among others [22]. It is defined as one of the leading causes of hospitalization for people above 65 years of age, with mortality rates between 5% and 15% once hospitalized [23].

To diagnose HF, one of the first tests performed is an X-ray, as it allows one to distinguish whether the patient's shortness of breath is of cardiac or pulmonary origin. In addition, when interpreting an X-ray, it is possible to identify certain parameters, such as pleural effusion and cardiomegaly, that suggest the presence of HF [24]. Studies have shown that with the help of AI and deep learning techniques, it is possible to achieve a highly accurate and assertive reading of the results, which facilitates the identification of cases of HF with great precision [25].

**Figure 3:**
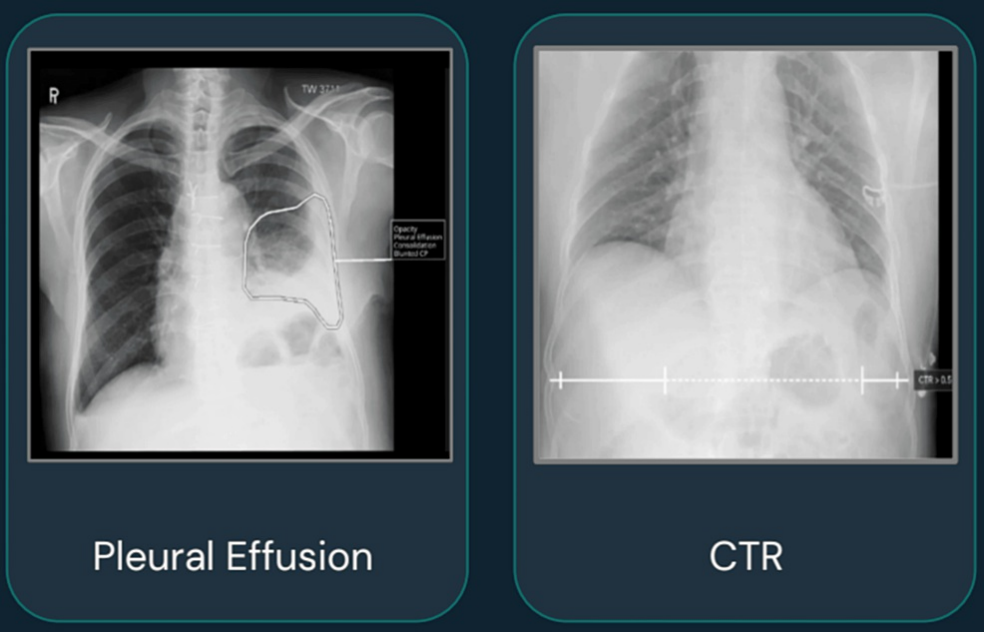
X-ray images with Qure.AI software in heart failure Image credits: Permission was obtained from the original publisher to adapt this image [16]. CTR: Cardiothoracic ratio.

Qure.AI’s AI algorithm, qXR-HF, facilitates the early detection of HF by analyzing chest X-rays for abnormalities. It can identify indicative patterns and features such as cardiomegaly, abnormal cardiothoracic ratio, and pleural effusion. These algorithms can process images in less than 60 seconds, enabling rapid and efficient diagnoses. Additionally, qXR-HF minimizes human error and enhances diagnostic accuracy, which is critical for HF, as early detection significantly improves treatment and recovery outcomes. Utilizing AI in HF detection also improves diagnostic precision and reduces the risk of misdiagnosis, thereby preventing treatment delays and potential medico-legal issues [3]. Figure 3 shows an image of a chest X-ray of an HF patient, in which Qure.AI’s software performed its analysis and clinical interpretation [16].

In a conducted study, the Qure.AI platform received anonymized chest X-rays from 10,100 patients to determine the presence of HF. Results showed 183 patients with increased cardiothoracic ratio (CTR) and pleural effusion. Out of the 106 patients who agreed to undergo further diagnostic tests, 77% were diagnosed with HF. In a comparison group of 106 randomly selected patients without these markers, 8% were diagnosed with HF. An accuracy of 84% was found in diagnosing HF using the chest X-ray images that were analyzed [9]. Detecting these parameters in X-rays can pose challenges to visual observation; therefore, the integration of AI markedly enhances diagnostic accuracy.

In a different Qure.AI project named "ART-IN-HF" involving 5,623 patients, the qXR tool demonstrated its capability to diagnose HF in individuals. Similar to the study previously mentioned, both cardiomegaly and pleural effusion were observed in 119 patients among the total number of the study’s participants. Out of these, 57 patients were contacted for further definitive HF diagnostic tests. HF was diagnosed in 49 out of 57 patients (86%). This study illustrated Qure.AI’s capacity to identify HF in its early stages rather than at advanced stages, resulting in improved outcomes for treated patients [14]. Prompt identification of various conditions is crucial in mitigating potential severe complications that could subsequently impact the patient's quality of life.

Employing AI for HF detection in chest X-rays holds promise for significantly enhancing diagnostic precision and swiftness. Harnessing the capabilities of AI algorithms enables healthcare practitioners to make more educated decisions and deliver optimal care to patients. As AI advancements progress, we can anticipate even more sophisticated applications in the diagnosis and management of HF and other medical conditions [3].

Among the articles reviewed, cases of pulmonary disease diagnosis using AI stand out as evidenced by the diagnosis of lung nodules through X-rays. Lung nodules are growths, typically around 3 cm in size, that develop in the lungs and can be classified as either benign or malignant, indicating the presence of cancer [26]. Their presence can be due to various causes, such as infections, scars, or malignant tumors. Patients often experience symptoms such as cough, possibly with blood, wheezing, and shortness of breath, among others [27].

These lesions can be classified as solid or mixed nodules, each exhibiting varying degrees of severity and growth rates [26]. Typically, lung nodules are detectable via chest X-rays or CT scans, after which additional tests can determine the appropriate course of action. For treatment, if the nodule is malignant, surgical removal is the optimal approach; conversely, if it is benign, a non-interventional approach may be considered as it may not adversely affect the patient [27].

Several studies have reported the use of deep learning algorithms for detecting health markers, producing promising outcomes. Qure.AI’s "qXR," can automatically detect and localize up to 29 markers, including those indicative of potential lung cancer. Features on chest radiographs, such as sharply circumscribed nodules or masses, irregular margins, and ill-defined lesions, can signal the presence of lung cancer. The qXR algorithm can accurately detect lung cancer nodules in less than a minute, also pinpointing their position and size. This tool assists clinicians in identifying tiny nodules that even experts might miss [28].

**Figure 4:**
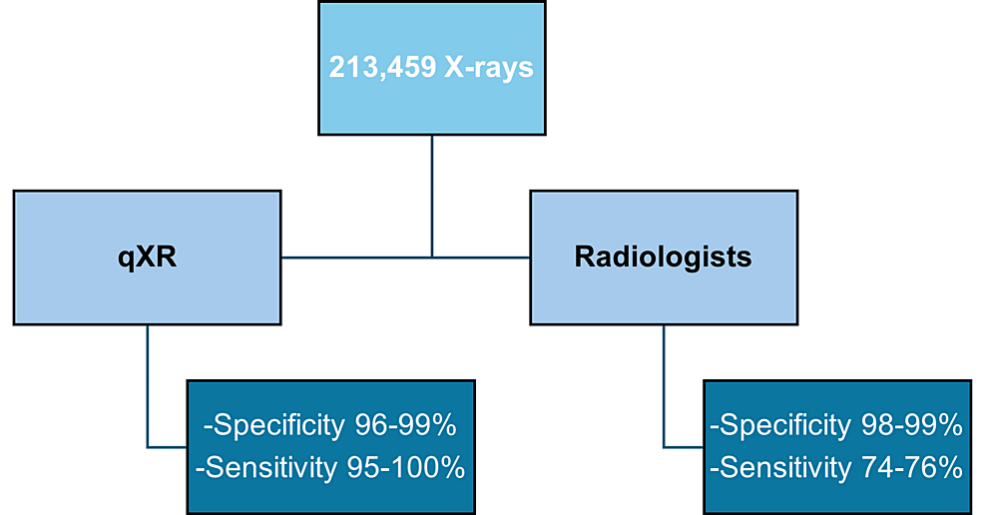
Comparison of results between Qure.AI's chest X-ray interpretation tool (qXR) and radiologists Image credits: This image was created by the authors.

In a study evaluating the performance of qXR in detecting malignant nodules, data were obtained from 213,459 randomly selected X-rays out of a total of 3.5 million. These X-rays were interpreted using the qXR tool and compared to radiologists’ reports. qXR demonstrated high accuracy in identifying nodules and even classified malignant nodules in the lungs. Qure.AI’s tool achieved a specificity of 96%-99% and a sensitivity of 95%-100%, while radiologists demonstrated a specificity of 98%-99% and a sensitivity of 74%-76% (Figure 4) [10].

**Figure 5:**
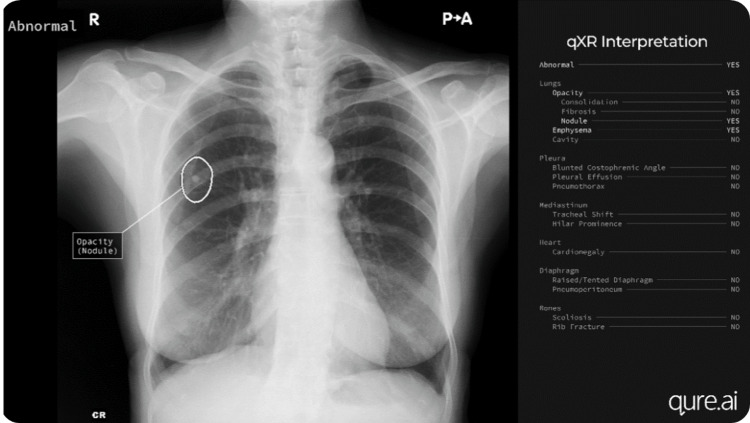
X-ray images with the use of Qure.AI software in lung nodules Image credits: Permission was obtained from the original publisher to adapt this image [29].

Figure 5 displays the result of qXR’s interpretation of a chest X-ray, showing the presence of a lung nodule, and illustrates how the tool generates the report [29].

A prospective multicenter quality improvement study was conducted to evaluate whether AI can be used as a chest X-ray screening tool in real clinical settings. It included a team of radiologists who integrated qXR into their routine workflow to analyze consecutive chest X-rays. During this period, a total of 65,604 chest X-rays were evaluated. The AI demonstrated strong overall performance in detecting both abnormal and normal chest X-rays, achieving a high negative predictive value of 98.9%. Additionally, a reduction of approximately 40.63% in turnaround time was observed from the pre-implementation to the post-implementation period of qXR [11].

The study concluded that qXR facilitated the exclusion of healthy individuals with a high degree of certainty, allowing radiologists to focus their attention on evaluating abnormalities in abnormal chest X-rays and the corresponding treatment strategies [11].

Before integrating evidence on the application of AI into routine clinical practices, it was imperative to undergo the approval process of various AI tools by the Food and Drug Administration (FDA). As of May 2021, a registry revealed that 141 algorithms had been approved. Additionally, the FDA classified each of these algorithms based on their level of risk (Table 2) [30].

**Table 2:** Risk level of algorithms approved in May 2021 by the FDA FDA: Food and Drug Administration.

Risk level	Number of approved algorithms
I	11
II	71
III	50
IV	9

In addition, Table 3 explains each risk level in relation to the implementation of these algorithms in medical software and their application in the clinical setting [31].

**Table 3:** Risk criteria for artificial intelligence algorithms

Level risk I	Level risk II	Level risk III	Level risk IV
The software provides information on low-impact conditions.	The software provides information on medium-impact conditions.	The software provides information on high-impact conditions.	The software provides information on very high-impact conditions.
Parameters such as heart rate, asthma attacks, and diagnostic options.	Parameters such as cardiovascular risks, 3D models, and diagnoses.	Parameters such as dose calculations, treatments, and photo analysis.	Parameters for diagnosing or treating critical illnesses.

Next, Table 4 shows some of the FDA-approved tools or algorithms and their respective uses [15,30,32,33].

**Table 4:** Artificial intelligence tools used in the clinical setting CSF: Cerebrospinal fluid; CT: Computed tomography; MRI: Magnetic resonance imaging; CTP: CT perfusion; MICA: Medical Imaging Cloud AI.

Product	Body part	Use	Risk
EyeArt	Eye	Identifies potential vision impairments or abnormalities	2
Quantib Brain 1.2	Brain	Estimates the proportion of gray matter and cerebrospinal fluid (CSF) in the brain.	2
WAVE Clinical Platform	Heart	Analyzes heart rate data to anticipate critical cardiac events.	3
Arterys Oncology DL	Lung and liver	Identifies tumors or other abnormalities in computed tomography (CT) and magnetic resonance imaging (MRI) scans.	3
Viz CTP	Brain	Assesses cerebral blood flow and overall brain health.	2
OsteoDetect	Bones	Predicts bone fractures based on X-ray images.	3
AI-Rad COMPANION	Lungs	Aids in diagnosing thoracic (chest) abnormalities using X-rays.	3
Arterys MICA	Heart	Analyzes MRI and CT scans of the heart to identify potential cancerous lesions.	3

## Conclusions

As time has progressed, AI has evolved into an invaluable tool in the clinical setting. As evidenced by the data gathered in clinical trials, this technology provides physicians with ease, speed, and precision in making diagnoses and selecting treatments. It is important to emphasize that the purpose of AI is to complement, not replace, the work of physicians. AI offers additional support to improve the effectiveness of diagnostic techniques, thereby achieving desirable outcomes. This technology is becoming an increasingly useful and sophisticated tool, which is capable of preventing future complications in patients by providing more accurate diagnoses and results from the outset.

Qure.AI is leading the way in applying AI to enhance global healthcare. They are dedicated to broadening their impact, creating new AI technologies, and collaborating with partners to address healthcare challenges. Their objective is to make top-quality healthcare accessible to everyone, everywhere through AI. Their cutting-edge solutions, meaningful case studies, strong partnerships, and regulatory achievements reinforce their status as a leader in AI-driven healthcare transformation. As Qure.AI continues to innovate, they have the potential to revolutionize healthcare delivery and greatly improve lives worldwide.
